# Primary effusion lymphoma in a patient with a good outcome on steroid alone treatment

**DOI:** 10.1002/ccr3.4020

**Published:** 2021-03-04

**Authors:** Ofir Koren, Ariel Aviv, Moran Avraham Kelbert, Ehud Rozner, Liza Lihtman, Elya Halfin, Yoav Turgeman

**Affiliations:** ^1^ Heart Institute Emek Medical Center Afula Israel; ^2^ Bruce Rappaport Faculty of Medicine Technion Israel Institute of Technology Haifa Israel; ^3^ Hematology Department Emek Medical Center Afula Israel; ^4^ Department of laboratory medicine Emek Medical Center Afula Israel

**Keywords:** human herpes virus type 8, human immunodeficiency virus; primary effusion, lymphoma; pericardial effusion, pleural effusion

## Abstract

Same clinical entity can have different biology and can behave differently. This must be kept in mind while making therapeutic decisions. Primary effusion lymphoma is a rare and devastating disease with high fatality. Chemotherapy provides limited benefit. We describe a unique case of a good outcome with steroid alone treatment.

## Graphical abstract

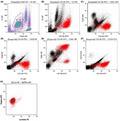

## 1 BACKGROUND

Primary effusion lymphoma (PEL) is rare, seen mostly in immunocompromised patients. Chemotherapy provides limited benefit, and the outcome is poor. We described PEL in patients who had good outcome on steroid alone treatment.

Primary effusion lymphoma (PEL) is a rare and distinct type of high‐grade non‐Hodgkin's B‐cell lymphoma (NHL).^1^ Previous reports have directly linked Human herpesvirus type 8 (HHV8) to PEL since the late 1990s.^2^

PEL is characterized by the presence of significant serous lymphomatous fluid collection in the pleural, pericardial, and peritoneal cavities without detectable solid tumor masses.^3^ It most often occurs in immunocompromised patients such as in the advanced stages of Acquired Immune Deficiency Syndrome (AIDS) ^4^ but has also been reported in immunocompetent elderly patients from HHV‐8 endemic areas such as central and southern Africa and the Mediterranean area. It typically involves middle‐aged patients with a significant male predominance of 6:1.^5^

The clinical scenario is directly related to the body cavity involved, the amount of effusion, and its interaction with the adjacent structures.^6^ The diagnosis is based on cytological, immunophenotypically, and viral characteristics of the involved effusion. PEL is suspected in the presence of diffuse large cells with abundant basophilic cytoplasm and irregular nuclei with prominent nucleoli resembling plasma cells.^7^ The lymphoma cells usually express the leukocyte origin marker CD45 but lack of the typical B‐ or T‐cell phenotype, while markers of plasma cell differentiation, CD38, and CD138 are usually present.^8^

To date, there is not a uniform treatment of PEL, and patients are generally treated with combined chemotherapy used for aggressive lymphomas known as the "CHOP" protocol, consisting of cyclophosphamide, hydroxydaunorubicin, oncovin (vincristine), and prednisone. Despite complete response rates of 43 to 57%, the median survival is still poor and estimated as six to nine months, due to early and usually refractory relapses.^9^

## 2 CASE PRESENTATION

An 84‐year‐old Jewish woman of North African ancestry presented to the emergency room complaining of effort‐related dyspnea, low‐grade fever, and dry cough for several days. Her past medical history includes only controlled hypertension. She uses Cinnarizine, Nifedipine, Aspirin, and Atenolol regularly.

**FIGURE 1:**
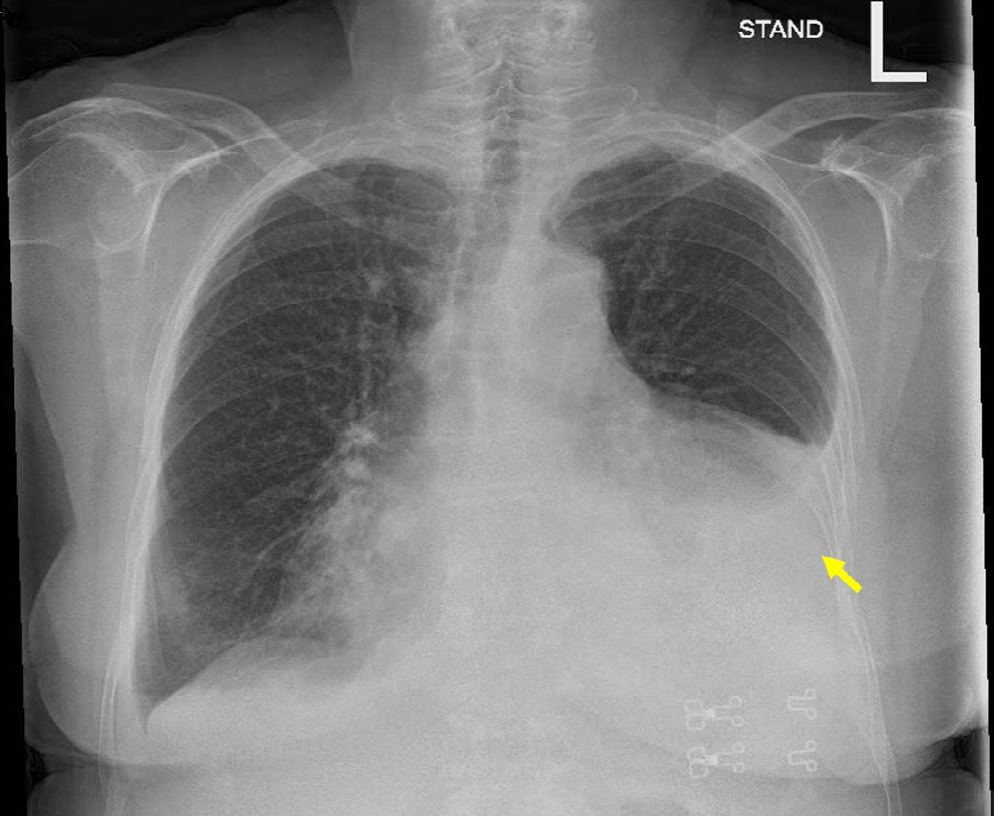
Chest X‐ray on admission shows an enlargement of the cardiac silhouette and large left pleural effusion (yellow arrow)

Upon arrival, a chest X‐ray (Figure 1) showed an enlarged heart silhouette and left lower lobe (LLL) infiltrate. Physical examination revealed diminished breath sounds and pleural dullness during percussion of the left hemithorax. After several days with no meaningful clinical improvement following treatment with azithromycin and ceftriaxone, CT angiography was performed indicating significant bilateral pleural effusion, moderate pericardial effusion, and no lymphadenopathy.

**FIGURE 2:**
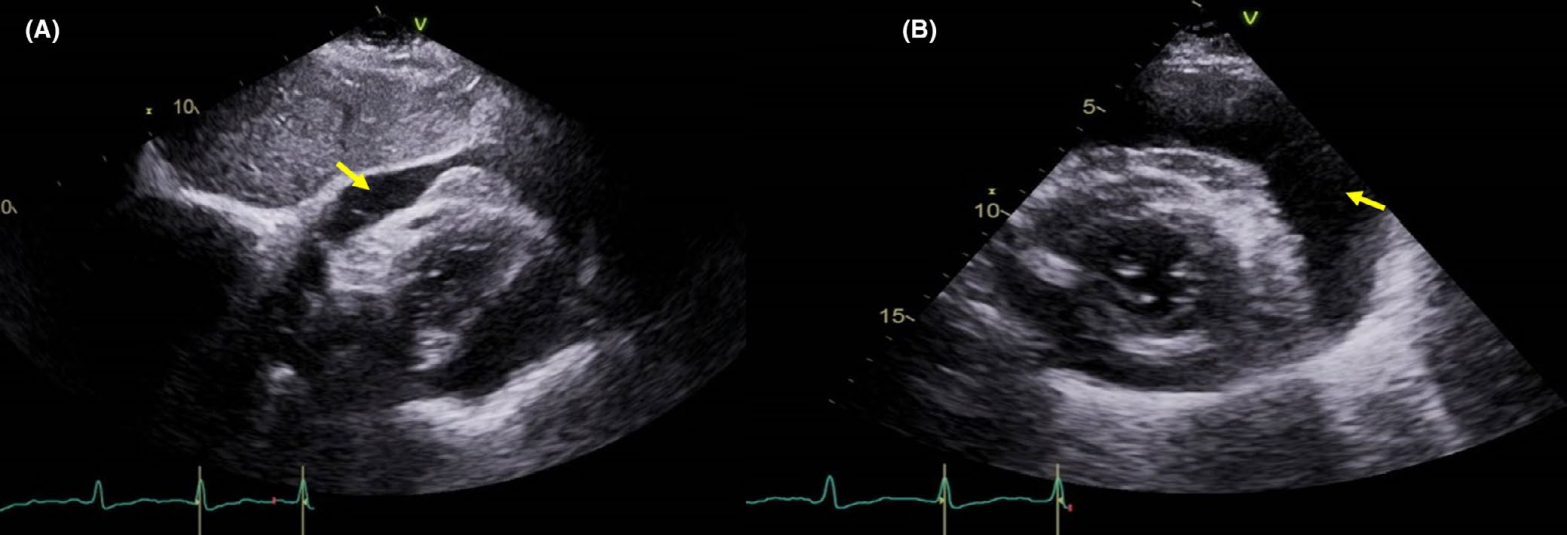
Transthoracic Echocardiography (TTE) on admission indicates large pericardial effusion (yellow arrow) seen on a subcostal view (A) and Short axis view (B)

Transthoracic echocardiography (Figure 2) showed a normal function of the left ventricle and a large volume of pericardial effusion with a diastolic right‐sided collapse. Urgent pericardiocentesis via a subcostal approach was performed, and 650 mL of serosanguinous fluid was withdrawn resulting in immediate relief of symptoms. Pericardial drainage continued for nine days using a pigtailed catheter. The patient underwent left pleurocentesis with the removal of 500 mL of exudative cloudy fluid. Pleural cell count indicated a total of 42 700 k/μL of white blood cells. Of them, 24% were monocytes, 13% basophils, and 63% were large unstained cells [LUC]. Pleural chemistry analysis showed a significant low glucose level (<11 mg/dL), high protein level [4.36 g/dL], low pH (6.99), and an extremely high LDH level (44 000 U/L) while Serum LDH was mildly elevated (640 U/L). The effusion to serum protein ratio was 0.71.

**FIGURE 3:**
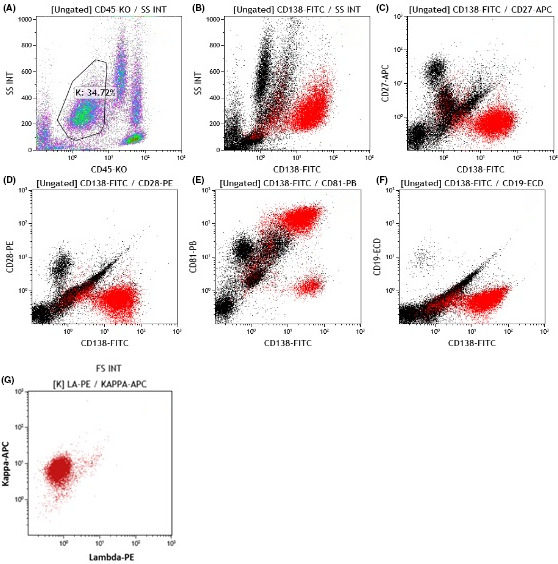
Eight color flow cytometric immunophenotyping of pericardial fluid indicated 34% CD45 negative cell population (A). Representative dot plots gated on CD45‐ (K gate) cells show high expression of CD138 (B), negative expression of CD27 (C), CD28 (D), CD19 (F), and a partial expression of CD81 (E). CD45‐ cells show positive monoclonality for Kappa light chain (G)

Flow cytometry (NAVIOS machine, Beckman Coulter), of the pericardial fluid, analyzed using the side scatter (SSC) gating, indicated 100 000 white blood cells. Of which, 25% were neutrophils (CD13+/CD10+), 25% monocytes (CD64+, CD123−, CD33+), and 12% were from the lymphoid lineage (CD20+, CD3+). An additional CD45− population comprised 38%‐45% of total cells with the following antigen properties: Kappa+, Lambda−, CD138+, CD38+, CD56+, CD81+, CD229+, CD74+, CD43+, CD19−, CD20−, CD24−, CD54−, CD27−, CD28−, CD200dim, CD117−, CD10−. CD81+ (Figure 3).

Microscopic examination of the fluid revealed a large immature atypical lymphocyte. Broad antigen analysis was performed indicating the following: HLA‐DR−, CD34−, CD123−, CD33−, CD64−, CD68−, CD4−, cyCd79a−, cyCD3−, MPO−, and TdT−.

**FIGURE 4:**
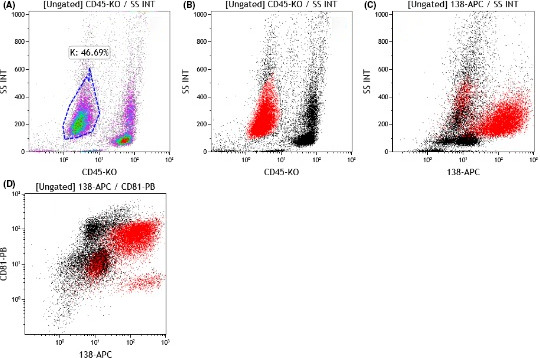
Eight color flow cytometric immunophenotyping of pleural fluid indicated 46% CD45 negative cell population (A and B). Representative dot plots gated on CD45‐ (K gate) cells show a high expression of CD138 (C) and a partial expression of CD81 (D)

Flow cytometry of the Pleural fluid showed the same antigenic properties (Figure 4). Immunofluorescent assays (IFA) using the lytic and latent IgG test for the detection of HHV‐8 were both positive. Human immunodeficiency virus (HIV) testing using the enzyme‐linked immunosorbent assay was negative.

A diagnosis of primary effusion lymphoma was established based on the cytologic findings. Suggestive of a lymphoid malignant infiltrate with cells positive for HHV8, along with the peculiar characteristic immunophenotype which was negative for classical B‐ and T‐cell markers, yet positive for plasma cell markers, that is, CD38 and CD138. Additionally, the lack of lymph node or spleen enlargement in whole‐body CT, and the lack of any mass at all were all consistent with the diagnosis of PEL.

After consulting with her family members, the patient chose not to start chemotherapy. Her treatment regimen consisted of antihypertensive medications (Nifedipine and Bisoprolol), 60 mg Prednisone which was slowly tapered over a course of several weeks, Ibuprofen (1800 mg daily for two weeks), 30mg Lansoprazole, and 100 mg Aspirin.

During the first six months after discharge, she underwent two successful pleural drains. At a two‐and‐a‐half‐year follow‐up, there were no adverse events other than a mild accumulation of pleural fluid that did not require drainage.

## 3 DISCUSSION

Primary effusion lymphoma is a rare lymphoma that usually occurs in immunosuppressed middle‐aged males. Recent reports also described PEL in immunocompetent HIV‐negative patients, mainly associated with HHV‐8 infection in endemic areas.

The differential diagnosis is wide and mostly includes inflammatory, metabolic, and infectious diseases that are well treated. The combination of serous fluid accumulation in various cavities without primary mass and unique cytological, immunophenotypically, and viral characteristics make it a definite diagnosis.

The most widely used protocol is the intensive chemotherapy combination known as the CHOP protocol, designed for aggressive lymphoma. It is accompanied by serious adverse side effects, a low remission rate, and a short duration of remission in those who do respond.

In most reports, the estimated life expectancy of immunocompromised PEL patients is of a few months, commonly reported as six to nine months. The reason probably relates to the progressive nature of the disease, patient comorbidities, compromised immune system status, procedural complications, failure of treatment, and adverse effects of chemotherapy.

Our case highlights an extremely rare scenario of PEL that occurred in an immunocompetent HIV‐negative elderly female in a non‐HHV8‐endemic area. PEL, as seen in our case, involved both the pleural and the pericardial spaces. Despite the patient's advanced age, her overall physical and cognitive functions were intact. The patient chose not to be treated with chemotherapy, and her sole treatment was prednisone. For 30 months, there were no adverse events. The patient is still alive and in complete remission and maintains excellent quality of life.

## 4 CONCLUSION

It seems that PEL has different clinical scenarios that we still do not fully understand affecting both immunocompetent and immunosuppressed patients, each with different epidemiologic features and a different outcome. The role of chemotherapy treatment in our case is still to be discovered as the patient decided not to abstain from active treatment. Physicians should be aware of all clinical scenarios to offer the best treatment option for their patients.

## CONFLICT OF INTEREST

The authors have no conflict of interest to declare.

## AUTHOR CONTRIBUTIONS

OK, AA, and MAK: contributed to the writing, editing, formatting of the main manuscript, and production of the figures. EP, LL, and YT: provided care to the patient and revised the manuscript. All authors have contributed and met the criteria for authorship.

## CONSENT FOR PUBLICATION

Written informed consent was obtained from the patient for publication of this case report and any accompanying images.

## Data Availability

The datasets used and/or analyzed during the current study are available from the corresponding author on reasonable request.
